# Quality Control of Widely Used Therapeutic Recombinant Proteins by a Novel Real-Time PCR Approach

**DOI:** 10.7508/ibj.2016.01.008

**Published:** 2016-01

**Authors:** Babak Mamnoon, Taghi Naserpour Farivar, Ahmad Reza Kamyab, Dariush Ilghari, Ali Khamesipour, Mohsen Karimi Arzenani

**Affiliations:** 1 Dept. of Molecular Medicine, Pasteur Institute of Iran, Tehran, Iran;; 2 Cellular and Molecular Research Center, Qazvin University of Medical Sciences, Qazvin, Iran;; 3 Dept. of Biology, Science and Research Branch, Islamic Azad University, Tehran, Iran;; 4 Center of Research and Training in Skin Diseases and Leprosy, Tehran University of Medical Sciences, Tehran, Iran

**Keywords:** DNA contamination, Real-time PCR, Streptokinase, Interferon-alpha (IFN-α)

## Abstract

**Background:**

Existence of bacterial host-cell DNA contamination in biopharmaceutical products is a potential risk factor for patients receiving these drugs. Hence, the quantity of contamination must be controlled under the regulatory standards. Although different methods such as hybridization assays have been employed to determine DNA impurities, these methods are labor intensive and rather expensive. In this study, a rapid real-time PCR test was served as a method of choice to quantify the *E. coli *host- cell DNA contamination in widely used recombinant streptokinase (rSK), and alpha interferon (IFN-α) preparations.

**Methods:**

A specific primer pair was designed to amplify a sequence inside the *E. coli *16S rRNA gene. Serial dilutions of DNA extracted from *E. coli *host cells, along with DNA extracted from Active Pharmaceutical Ingredients of rSK, and IFN-α samples were subjected to an optimized real-time PCR assay based on SYBR Green chemistry.

**Results:**

The test enabled us to detect a small quantity of genomic DNA contamination as low as 0.0002 pg in recombinant protein-based drugs. For the first time, this study showed that DNA contamination in rSK and IFN-α preparation manufactured in Pasteur Institute of Iran is much lower than the safety limit suggested by the US FDA.

**Conclusion:**

Real-time PCR is a reliable test for rapid detection of host-cell DNA contamination, which is a major impurity of therapeutic recombinant proteins to keep manufacturers’ minds on refining drugs, and provides consumers with safer biopharmaceuticals.

## INTRODUCTION

Therapeutic recombinant products are extensively used for human medical purposes. Recombinant proteins can be expressed in various types of living organisms^[^^1^^,^^2^^]^. *Escherichia coli* is one of the most commonly used host cells for the manufacture of biopharmaceuticals due to its well-characterized genetic map^[^^3^^-^^5^^].^ Although using *E. coli* for the production of therapeutics has many advantages, contamination of biopharmaceuticals with bacterial DNA has become a major concern for manufacturers. Genomic DNA contamination in biopharmaceuticals has been considered as a possible risk factor^[^^6^^]^. It has been supposed that DNA contaminants may be integrated into the genome of patient’s recipient cells and express a new foreign gene or alter the level of gene expression^[^^7^^]^. 

It is, therefore, crucial for biopharmaceutical industries to monitor DNA contamination^[^^8^^]^ and keep the quantity of impurities in bioproducts as low as a safety limit suggested by the regulatory authorities such as FDA, which must be lower than 100 pg of DNA per milligram of protein^[^^9^^]^. Generally, the hybridization assay and total DNA threshold assay have been used for the quantitative detection of host cell DNA in pharmaceuticals; however, these methods are time-consuming and expensive^[^^10^^]^. 

In recent years, great interests have been focused on real-time PCR assay as a new method for simultaneous detection and quantification of PCR products^[^^11^^]^. Real-time PCR has been employed for detection of bacterial, and viral loads in therapeutic proteins^[^^12^^-^^16^^]^. In this study, we established a novel sensitive real-time PCR test based on SYBR Green chemistry to detect a small quantity of *E. coli* host-cell DNA contamination in rSK and IFN-α samples produced at Pasteur Institute of Iran. Streptokinase and interferon are protein-based drugs produced in *E. coli* host cells^[^^17^^,^^18^^]^. Streptokinase is used as an intravenous thrombolytic agent for treatment of acute myocardial infarction^[^^19^^]^. Interferon acts as an integral part of the innate immune response and targets specific cell-surface receptors^[^^20^^]^, which are conducive to the interferon-mediated antiviral response^[^^21^^]^.

## MATERIALS AND METHODS

**Cell culture**

*E. coli* W3110 strain was provided from Pasteur Institute of Iran. As the first step of normalization, a single colony of the cells was used to inoculate into 5 ml of Luria-Bertani broth. The colony was incubated at 37ºC overnight with shaking at 250 rpm until the optical density at 600 nm reached 3.0, which was approximately equivalent to 2.4×10^9^ cells/ml (Perkin-Elmer Lambda UV/visible Spectrophotometer, Applied Biosystems, UK).

**Preparation of** ***E. coli*** **genomic DNA**

Cells were precipitated by centrifugation at 17226 ×g for 2 min. Genomic DNA was isolated using DNeasy Blood and Tissue Kit (Qiagen, Germany) according to the manufacturer’s protocol. At the second step of normalization, Optical density (260/280 nm and 260/230 nm) and concentration were measured using a NanoPhotometer 2000c (Thermo Science, USA).

**Primer design and PCR test**

In this study, four sets of forward and reverse primers were designed for the conserved region of the eubacteria 16S rRNA gene using Primer Express software (Foster City, USA). The sequence information is found in the NCBI GenBank database (Accession Number: J01859). The specific oligonucleotide primers were designed for the conserved region of 16S rRNA gene (Table 1), and primer pairs were synthesized by Bioneer Corporation (Korea). All primer pairs were used in different PCR runs from the viewpoint of primer dimmer formation. Among the designed primers, the primer pair 16S_2 _displayed higher specificity and efficiency with minimal primer pair formation.

PCR reaction was carried out in a Mastercycler Personal (Eppendorf, Germany). The cycle parameters were as follows: initial heat denaturation at 94ºC for 1 min, followed by 35 cycles of denaturation at 94ºC for 20 s, annealing at 60ºC for 30 s, and extension at 72ºC for 30 s. To ensure the complete extension, reaction mixture was incubated at 72ºC for 5 min. Genomic DNA (10 ng) was amplified in a total volume of 20 µl mixture containing 10 pM each of forward and reverse primer (0.5 µl), 10 µl Ex Taq Master Mix 2× (Takara, Japan, and 8 µl nuclease free water. PCR products were analyzed by electrophoresis on 1.5% agarose gel (Sigma, USA).

**Optimization of real-time PCR**

In order to normalize and calculate primer efficiency, different concentrations of primer pairs were assessed. In this assay, SYBR Green I was used as a fluorescent dye. Real-time PCR reactions were carried out via an ABI 7300 real-time PCR instrument (Applied Biosystems, USA).

Amplification efficiency was validated using five-fold serial dilutions of DNA template as 2000, 400, 80, 16, 3.2, 0.64, 0.128, 0.025, 0.005, 0.001, and 0.0002 pg. Standard curve was drawn by plotting the logarithmic input DNA concentration versus mean cycle of threshold (mCt). Subsequently, the slope of standard curve was determined and PCR efficiency was calculated using the formula; E = (10^[-1/slope]^-1)× 100.

Optimum reaction condition in a total volume of 20 µl was obtained with 10 pM each forward and reverse primer (0.5 µl), 10 µl SYBR Green PCR Master Mix 2× (Takara, Japan) containing Taq DNA polymerase, SYBR Green I, Tris-HCl, MgCl_2_, KCl, and deoxynucleotide triphosphate, 0.4 µl ROX dye (6-Carboxy-x-rhodamine), 7.6 µl nuclease-free water, and 1 µl template genomic DNA. Thermal cycling program was performed with an initial heat denaturation at 95˚C for 1 min, followed by 40 cycles of denaturation at 95˚C for 20 s, and combined annealing/extension at 60˚C for 35 s and 95ºC for 15 s, 60ºC for 1 min and 95ºC for 15 s. In the melting stage, temperatures were raised 0.5ºC for each step. Dissociation stage was performed after the amplification stage to verify the specificity of the PCR products. Each amplicon was identified by its specific melting curve Tm. A reaction including PCR Master Mix and nuclease-free water was used as non-template control in each experiment. All reactions were run in duplicate. 

**Table 1:** Sequences of oligonucleotide primer pairs

**Forward primer**	**Reverse primer**	**Nucleotide position**	**product ** **size (bp)**
16S-F1 AGAAGCTTGCTCTTTGCTGA	16S-R1 CTTTGGTCTTGCGACGTTAT	78-197	120
16S-F2 AAAGGAGACTGCCAGTGATA	16S-R2 AGGTCGCTTCTCTTTGTATG	1149-1263	115
16S-F3 CATTGACGTTACCCGCAGAA	16S-R3 CGCTTTACGCCCAGTAATTCC	476-576	101
16S-F4 CCATGAAGTCGGAATCGCTAG	16S-R4 ACTCCCATGGTGTGACGG	1326-1419	94

**DNA extraction from therapeutic proteins**

Due to the presence of inhibitory agents in final products, DNA was extracted from Active Pharmaceutical Ingredients of rSK and IFN-α samples. To achieve this purpose, five batches of each drug, which were produced for five successive weeks, were selected for DNA purification. After phenol/ chloroform extraction, the supernatant was transferred into a microcentrifuge tube. Then, DNA was precipitated using 400 µl isopropanol. After washing twice with 700 µl ethanol, DNA pellets were suspended in nuclease-free water.

**Detection of DNA contamination**

DNA templates, including 1 µl each serially diluted *E. coli* genomic DNA, 1 µl DNA extracted from rSK, and 1 µl DNA extracted from IFN-α were subjected to real-time PCR test. The total volume of each reaction mixture was 20 µl containing 10 pM each forward and reverse primer (0.5 µl), SYBR Green PCR Master Mix 2 × (10 µl), ROX dye (0.4 µl), nuclease-free water (7.6 µl), and 1 µl DNA template. All reactions were run in duplicate, and non-template controls were added to the experiment. Cycling conditions were hold at 95ºC for 1 min, followed by 40 cycles at 95ºC for 20 s, and 60ºC for 35s. PCR products were visualized on 1.5% agarose gel.

**Validation of real-time PCR**

According to the FDA guidelines for industry, namely bioanalytical method, validation, the reproducibility, precision, linearity, and detection limit of the real-time PCR assay were validated. *E. coli *genomic DNA (100 µg) was fragmented using *Hind*III and *Eco*RI enzymes, and fragmented DNA was used as the standard DNA. The reproducibility and precision were measured using 50 and 100 pg of *E. coli* genomic DNA (gDNA), and the concentration of samples were measured three times on different days. The mCt values, and average of standard deviations (SD) were also calculated.

For detection of linearity, standard samples containing 2000, 400, 80, 16, 3.2, 0.64, 0.128, 0.025, 0.005, 0.001, and 0.0002 pg of *E. coli* gDNA, along with negative samples, were prepared. The concentrations of samples were obtained three times on different days. Standard curves were plotted for each experiment, and the correlation coefficient was calculated using least square method.

The detection limit was evaluated using minimum level of concentration at which the samples can be detected. Standard samples containing 2000 to 0.0002 pg of genomic DNA, along with negative solutions, were used for the validation of real-time PCR assay.

## RESULTS

**Amplification of DNA extracted from rSK and** **IFN-α**

**Fig. 1:**
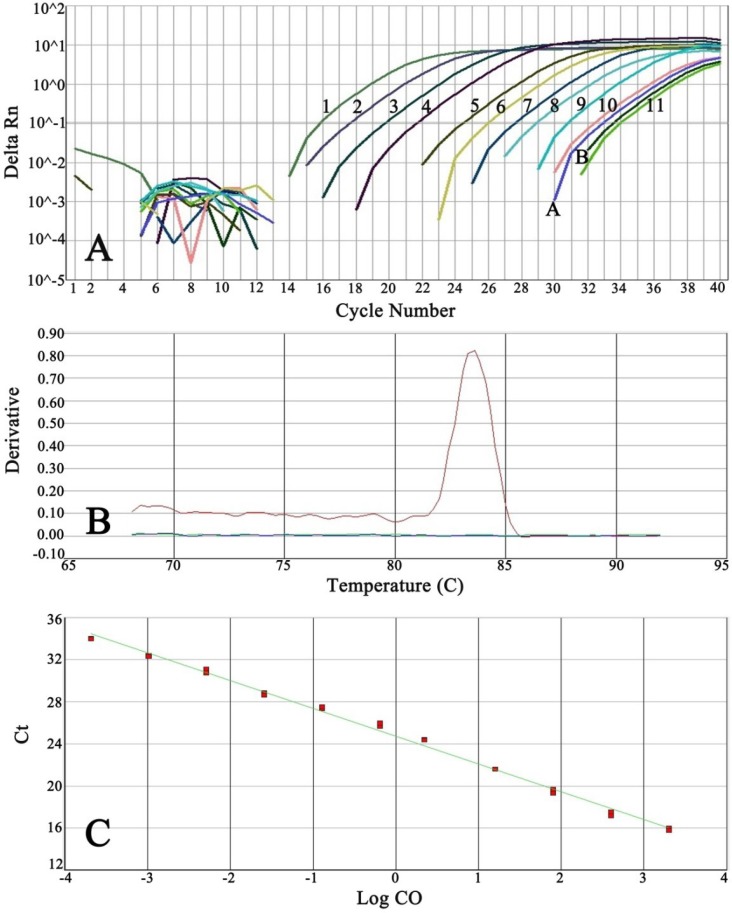
Specificity of real-time PCR test for the quantity of DNA contamination in biopharmaceuticals; (A) Amplification plot of 5-fold serial diluted *E. coli* genomic DNA concentrations along with the amplification plot of DNA extracted from therapeutic proteins; 1, 2000 pg; 2, 400 pg; 3, 80 pg; 4, 16 pg; 5, 3.2 pg; 6, 0.64 pg; 0.0128 pg; 8, 0.025 pg; 9, 0.005 pg; 10. 0.001 pg; 11. 0.0002 pg; A, Alpha interferon; B, Streptokinase; (B) Melting curve analysis of PCR products; (C) Standard curve

**Fig. 2:**
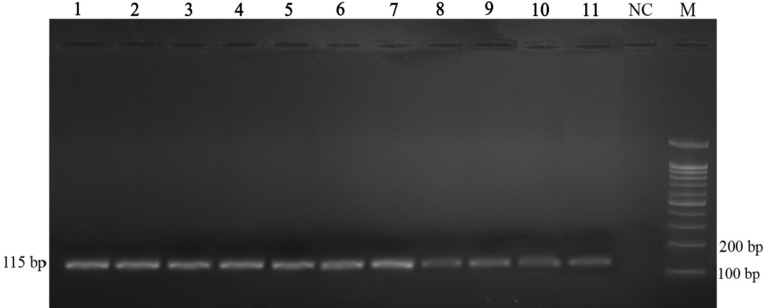
Gel electrophoresis of PCR products. Lane 1, 2000 pg; Lane 2, 400 pg; Lane 3, 80 pg; Lane 4, 16 pg; Lane 5, 3.2 pg; Lane 6, 0.64 pg; Lane 7, 0.128 pg; Lane 8, 0.025 pg; Lane 9; 0.005 pg, Lane 10; 0.001 pg, Lane 11; 0.0002 pg, NC; negative control, M; marker.

Serially five-fold dilution of *E. coli* genomic DNA from 2000 to 0.0002 pg, one pair of negative controls, and DNA extracted from rSK and IFN-α preparations were run in duplicate (Fig. 1A). Melting curve analysis of PCR products did not show any primer-dimer formation (Fig. 1B). Moreover, this graph demonstrated melting of amplification fragment at 83.9ºC. The post-PCR analysis confirmed and showed the target fragment (115 bp) without non-specific products (Fig. 2).

The slope of standard curve, y-intercept, determination of coefficient (R^2^), and PCR efficiency were obtained as -2.63, 25.64, 0.99, and 100%, respectively. Standard curve presented Ct values (mean ± SD) between 15.58 ± 0.163 and 34.03 ± 0.037 (Fig. 1C).

**Quantitative detection of DNA** **contamination**

The mean of two Ct values (mCt) obtained from duplicate amplification of DNA extracted from each rSK and IFN-α was calculated separately. The mCt values (32.89 and 33.28 for rSK and IFN-α, respectively) were put instead of “Y” values in the standard curve equation (Y = -2.63X + 24.72) to calculate “X” values. After getting the common logarithm of “X” values, the estimated amounts of *E. coli* host-cell DNA contamination in rSK and IFN-α preparation were 0.0012 and 0.0017 pg per mg of products, respectively (Table 2). The values calculated from the experiments were much lower than the limit permitted by the FDA^[^^9^^]^.

**Validation of real-time PCR**

In the present study, some quality control procedures, including the reproducibility, precision, detection limit, and linearity of real-time PCR method were performed. The reproducibility was determined by calculating the mCt values as 19.17, 19.64, and 18.88 for 50 pg of standard DNA, and 20.33, 20.44, and 20.12 for 100 pg of standard DNA on different days (Table 3). The precision was evaluated as 50.67 ±1.28 and 101.12 ± 1.44 for 50 and 100 pg of standard DNA, respectively (Table 4). The linearity was calculated by a standard curve using the least square method. The mean value of the correlation coefficient obtained from three experiments was 0.99. 

**Table 2:** DNA content of recombinant protein-based drugs

**Recombinant ** **protein**	**Drug concentration** **(mg/ml)**	**DNA content** **(pg/mg of product)**
rSK	1.2	0.0012
IFN-α	1.1	0.0017

The detection limit of the assay was 0.0002 pg of DNA. The range of the standard curve was from 0.0002 to 2000 pg of DNA.

## DISCUSSION

Therapeutic recombinant proteins are widely used for human therapies. Host-cell genomic DNA contamination in biopharmaceuticals is a potential risk factor for consumers. Therefore, FDA and other regulatory agencies have defined specific quality control and safety criteria, which manufacturers are required to quantify DNA impurities in recombinant therapeutics at different stages of production^[^22^,^^23^^]^. To detect the minimum amount of impurities, the method involved in the quantitative detection of DNA contamination must be accurate and sensitive enough.

For a few years, radioactive-based expensive methods, including the species-specific DNA hybridization assay, have been used to quantify bacterial host-cell DNA contamination in biopharma-ceuticals [10]. Ji *et al*.^[^^24^^]^, reported a safe non-radioactive slot-blot hybridization assay to quantify *E. coli* DNA contamination levels in purified protein-based drugs with a sensitivity to detect 2.5pg *E.coli* DNA. Afterwards, Gregory *et al*.^[^^25^^]^ described a safer and less-expensive alternative method, i.e. PCR amplification and dioxygenin labeling of the genes encoding 5S rRNA followed by an affinity-based collection, for detection of 1 pg/mL of the extracted *E.coli* genomic DNA^[^^25^^]^. Although the Gregory's method seemed sensitive, it was capital-intensive to be applied routinely in the biopharmaceutical industries. This made researchers to search for even more inexpensive assays that might be applied readily by manufacturers. Real-time PCR has recently been reported as an alternative method for simultaneous detection and quantification of DNA sequence in manufactured products.In this study, a novel real-time PCR test, based on SYBR Green chemistry, was established for the precise measurement of *E. coli *host-cell DNA contamination in two widely used recombinant protein-based drugs, i.e. rSK and IFN-α. Despite the fact that real-time PCR has many privileges, it has not been employed routinely in the pharmaceutical industries, mainly because real-time PCR reactions may be inhibited by various agents that are present in biopharmaceuticals^[^^26^^]^. In this experiment, to eliminate the inhibitory effect of the agents, *E. coli* genomic DNA was first extracted from the Active Pharmaceutical Ingredients of biopharmaceuticals and then was subjected to real-time PCR test. Using this method, we are able to detect reliably a very small amount of bacterial genomic DNA. The gene copy number was calculated according to the molecular mass of the *E. coli *genome, namely 2.86×10^9^ Da or 4.7491×10^-3^ pg. There are seven genomic copies of the 16S rRNA gene in each bacterium^[^^27^^]^, and the molecular mass of each copy is 6.79×10^-4^ pg. Thus, the sensitivity of our assay was 0.0002 pg equivalent to 1 copy of the 16S rRNA gene (equivalent to one *E. coli* bacterium). Although some kinds of probes, including those labeled with fluorescent dyes, can significantly enhance the specificity of real-time PCR^[^^28^^,^^29^^]^, we exploited SYBR Green chemistry to establish an inexpensive detection assay. It is known that primer-dimer formation is one of the major problems using SYBR Green dye. To avoid this problem, we optimized the real-time PCR reaction condition by designing accurate primers as well as by finding the most effective salt concentration (4 mM), primer concentration (10 pM), optimal annealing temperature and time (60ºC for 35 s).

**Table 3.** The reproducibility of assay

**Concentration of standard** **DNA (pg)**		**mCt**	
	**Day 1**	**Day 2**	**Day 3**
50	19.17	19.64	18.88
100	20.33	20.44	20.12
			

**Table 4:** The precision of assay

**Concentration of standard DNA (pg)**	**Concentration of calculated DNA (mean ± SD)**
50	50.67 ± 1.28
100	101.12 ± 1.44

In conclusion, we have succeeded to establish a new real-time PCR approach based on SYBR Green chemistry for measurement of *E. coli *genomic DNA impurities in biopharmaceuticals, with a detection limit of 0.0002 pg DNA per mg of therapeutic recombinant proteins. The present study show that *E. coli* host-cell DNA contamination in rSK and IFN-α manufactured at Pasteur Institute of Iran is much lower than the safety limit suggested by FDA.
